# Response to “Weaning order of vasoactive drugs”

**DOI:** 10.1186/s13054-018-2255-y

**Published:** 2019-03-12

**Authors:** Kyeongman Jeon, Jae-Uk Song, Gee Young Suh

**Affiliations:** 1 Department of Critical Care Medicine, Samsung Medical Center, Sungkyunkwan University School of Medicine, Seoul, Republic of Korea; 2 Division of Pulmonary and Critical Care Medicine, Department of Medicine, Samsung Medical Center, Sungkyunkwan University School of Medicine, 81 Irwon-ro, Gangnam-gu, Seoul, 06351 Republic of Korea; 3 Division of Pulmonary and Critical Care Medicine, Department of Medicine, Kangbuk Samsung Hospital, Sungkyunkwan University School of Medicine, Seoul, Republic of Korea

We thank Drs Michels and Trevisol for their thoughtful comments [1] regarding our recent randomized controlled trial on the incidence of hypotension while tapering vasopressors in patients on concomitant norepinephrine (NE) and vasopressin (AVP) recovering from septic shock [2].

We understand their concern that the rapid taper rates of NE in our study could have influenced the hemodynamic tolerance to the vasoactive drug tapering. As they stated in their letter, this could be supported by the fact that the median time to hypotension after tapering vasopressor was shorter in the NE-tapered-first group (2.0 (1.2–2.5) h) than the AVP-tapered-first group (4.3 (2.5–5.1) h). In this study, because we tried to taper the same relative amount of both vasopressors (33%), we ended up decreasing NE by 0.1 μg/kg/min which is a relatively large dose compared with conventional doses of 2–5 μg/min [3]. However, the best method of weaning NE including magnitude as well as time interval is not established and should be a subject for future studies [4].

Drs Michels and Trevisol seem to have a misunderstanding about Table 3 in our original manuscript [2], which compares clinical characteristics of the subgroup of patients with hypotension according to which vasopressor was tapered immediately before developing the primary outcome, regardless of treatment allocation. The hospital mortality in patients developing hypotension after NE tapering was 46.5%, not 58.97%. The high hospital mortality seen in these patients despite a short duration of vasopressor support until study inclusion may be due to the fact that, at inclusion, these patients were still severely ill patients with severe shock needing support by two pressors at high doses. Also, intensive care unit (ICU) mortality was 28.3%, which suggests that at least some of the patients (15.2%) died after patients were transferred to the general ward, presumably due to his or her underlying conditions and not due to the episode of septic shock that brought them to the ICU. The duration from the maximum dose of NE to the initiation of intervention could have provided more information on patient characteristics. Unfortunately, these data could not be extracted from our case report forms.

Drs Michels and Trevisol also suggest that we should have administered AVP at the maximum dose of 0.04 U/min before tapering and that the administration of corticosteroid and dobutamine might affected the outcome. Our hemodynamic resuscitation protocol in the management of sepsis was in line with the Surviving Sepsis Campaign Guideline [5], which suggests using up to 0.03 U/min of AVP and corticosteroid infusion if adequate fluid resuscitation and NE therapy are able to restore hemodynamic stability. The majority of our patients received corticosteroid treatment using the protocol described in the Methods section of our original manuscript [2]. However, dobutamine was used in only six (8%) patients. Therefore, we do not think the administration of either corticosteroids or dobutamine had significant influence on the outcome.

## Abbreviations

AVP: Vasopressin
NE: Norepinephrine
